# Surgical Reports

**Published:** 1857-07

**Authors:** 


*Surgical Reports.*—With our present number we commence a series of clinical reports of cases treated under the care of Professor Hamilton, at the Hospital of the Sisters of Charity, by Dr. B. H. Lemon, clinical assistant. These reports are intended as companions to the medical cases reported by Dr. A. W. Nichols. The two series together will constitute a valuable practical repertory. They are made up from cases presented to the class of the University of Buffalo during the last session, and will afford some index to the clinical advantages of that school.

